# Information Theory in Game Theory

**DOI:** 10.3390/e20110817

**Published:** 2018-10-24

**Authors:** MHR Khouzani, Pasquale Malacaria

**Affiliations:** School of Electronic Engineering and Computer Science, Queen Mary University of London, Mile End Road, London E1 4NS, UK

**Keywords:** information theory, game theory

Information theory, as the mathematics of communication and storage of information, and game theory, as the mathematics of adversarial and cooperative strategic behaviour, are each successful fields of research on their own. Both disciplines have a far-reaching impact on modern science and society: Information theory underpins the creation and the engineering of the digital age; game theory informs economics and social sciences. Moreover, it is now becoming increasingly apparent that they have a potentially large synergy, and several problems can be naturally studied at the intersection of information theory and game theory.

Some of these problems are explored in this special issue. The contributions include works in computer security, foundations of game theory, statistical mechanics, networks, behavioural sciences, healthcare and business processes.

In the field of computer security, for example, one agent wants to minimize the leakage of information in an adversarial setting. The subject of study of [1] is leakage games, where the interplay between defender and attacker is modelled in a game-theoretic framework where the payoff is information leakage. The authors explore several games in this framework, e.g., where players act simultaneously or sequentially, and the choices of the defender may be visible or not visible to the attacker. The authors prove that in this setting some subtle differences from classical game theory appear, for example, equivalent strategies in classical game theory are here distinct. Multi-objective game theory is applied in [2] to the password security problem and it introduces different concepts of entropy to measure the quality of a password choice process: The memorability of the password is measured by Shannon entropy, while the difficulty for the attacker of guessing it is measured by min-entropy. The problem of optimal channel design is studied using game theory in [3]: This is the problem of designing a (probabilistic) channel whose output leaks minimally from its input, for a given input probability distribution and for hard and soft design constraints. The problem is translated in a game theoretical framework where the Nash equilibria for the defender are shown to be the solution of a convex optimization for the optimal information theoretical channel.

An analysis of the fundamental concept of solution in games is the subject of [4]. Starting from recent algorithmic results establishing that Nash equilibria are computationally equivalent to fixed points, the authors propose a new class of universal non-equilibrium solution concepts arising from an important theorem in the topology of dynamical systems: Chain recurrent sets. Kullback–Leibler divergence is used in the study of these solution concepts.

Evolutionary game theory and entropy are used in [5] in the field of statistical mechanics: The paper investigates the stabilizing effect of entropy in a coordination game with five strategies where the subgames of the first two and the last three strategies are identical to a ferromagnetic Ising and a three-state Potts model, respectively.

A well known research area where game theory (both adversarial and cooperative) and information theory fruitfully interact is in the study of communication networks: In [6], an incentive mechanism is designed for optimal network traffic offloading. The offloading problem is formulated as a Stackelberg game: The macro cell base station is the leader and small cells are followers. The equilibrium of the game is proved to exist and to be unique. Shi et al. [7] introduces a novel Nash Bargaining Solution (NBS)-based cooperative game-theoretic framework for power control in a distributed multiple-radar architecture underlying a wireless communication system. Li et al. [8] proposes two-layer coalition-auction game-based transaction (CAGT) mechanism to optimize the performance of 5G networks.

A classical topic in game theory is the study of iterated games, one of the famous contributions being Axelrod’s tournaments. The role of information in iterated games is explored in [9]. The work investigates experimental behavioral data about rhesus monkeys playing thousands of “matching pennies” games. An information theoretical analysis of the experiments indicates that the monkeys extract non-Markovian information, i.e., information from more than just the most recent state of the game.

This special issue includes also two interesting applications: The first is in healthcare [10], where coalition games and information theory are used to enhance the accuracy of false alarm detection in Intensive Care Units. In the second application [11], a “Co-Opetitive” (cooperative-competitive) game theoretical framework is developed for supply chain business processes involving competitor teams cooperating on some parts of the process. The paper develops an automated negotiation model both for the collaborative game process among the team members and for the competitive negotiation process.
